# Pneumothorax detection with thoracic ultrasound as the method of choice in interventional pulmonology - A retrospective single-center analysis and experience

**DOI:** 10.1186/s12890-023-02511-7

**Published:** 2023-06-26

**Authors:** Sonja Sieber, Jakob Garbe, Sebastian Böhm, Stephan Eisenmann

**Affiliations:** 1 Department of Internal Medicine, University Hospital Halle (Saale), Ernst-Grube-Straße 40, 06120 Halle, Germany; 2 Department of Internal Medicine, University Hospital Tübingen, Otfried-Müller-Straße 10, 72076 Tübingen, Germany

**Keywords:** Thoracic ultrasound, Lung ultrasound, Pneumothorax, Interventional pulmonology

## Abstract

**Background:**

Recent studies have shown that thoracic ultrasound (TUS) is not inferior to chest radiography (CR) in detecting pneumothorax (PTX). It is unclear if adopting TUS can reduce the number of CR in the daily clinical routine. This retrospective study investigates the utilization of post-interventional CR and TUS for PTX detection after the introduction of TUS as the method of choice in an interventional pulmonology unit.

**Methods:**

All interventions with CR or TUS for ruling out PTX performed in the Pneumology Department of the University Hospital Halle (Germany) 2014 to 2020 were included. The documented TUS and CR performed before (period A) and after the introduction of TUS as the method of choice (period B), as well as the number of diagnosed and missed PTX were recorded.

**Results:**

The study included 754 interventions (110 in period A and 644 in period B). The proportion of CR decreased from 98.2% (n = 108) to 25.8% (n = 166) (p < 0.001). During period B, a total of 29 (4.5%) PTX were diagnosed. Of these, 28 (96.6%) were detected on initial imaging (14 by CR, 14 by TUS ). One PTX (0.2%) was initially missed by TUS, none by CR. Confirmatory investigations were ordered more frequently after TUS (21 of 478, 4.4%) than after CR (3 of 166, 1.8%).

**Conclusion:**

The use of TUS in interventional pulmonology can effectively reduce the number of CR and thus save resources. However, CR may still be favored in specific circumstances or if pre-existing conditions limit sonographic findings.

## Background

Pneumothorax (PTX) is a potentially life-threatening condition which needs to be taken into account in a variety of settings, including pulmonary care, intensive care, emergency medicine and surgery [1]. Especially iatrogenic PTX that is commonly linked to various procedures in interventional pulmonology is a frequent complication and should be promptly and safely detected [2, 3]. PTX is diagnosed using different imaging methods, most frequently chest radiography (CR) and – more recently – thoracic ultrasound (TUS). Studies revealed a similar specificity of TUS and CR for the detection of PTX but have reported a higher sensitivity for PTX exclusion when performed by TUS [4–6]. Comparative diagnostic studies were often conducted in a trauma setting or intensive care unit (ICU) and – less frequently – in interventional pulmonology. Here, TUS was reported to be feasible and safe when compared to CR after transthoracic biopsies, transbronchial biopsies and transbronchial cryobiopsies [7–16].

Despite this evidence, the adoption of TUS for PTX exclusion in daily practice is slow, and hindrances to widespread adoption are unclear or have not yet been investigated [17]. Furthermore, the effect of TUS introduction in routine care is largely unknown: While a reduction in CR was observed in an ICU setting [18], it is unknown whether these findings can be generalized to other fields of care. Especially in interventional pulmonology, conditions such as (partial) pleurodesis, bullae or contusions may limit the conclusiveness of TUS for PTX detection when compared to the ICU setting [12]. Our retrospective observational study aims to evaluate if using TUS as the method of choice for ruling out PTX can effectively reduce the number of CRs in the routine care of an interventional pulmonology unit.

## Methods

This study was conducted as a retrospective single-center observational study in the Pulmonology Department of the University Hospital Halle (Saale), Germany. The Institutional Review Board of the Martin-Luther-University Halle-Wittenberg approved the investigation (IRB number: 2021 − 149, July 21, 2021).

### Study design

All pulmonary interventions (bronchoscopy and ultrasound) in adult patients with subsequent PTX exclusion by CR or TUS performed between January 2014 to December 2020 were included. Until March 2017, post-interventional CR was the method of choice for PTX detection (period A). In April 2017, a new interventional pulmonology team was introduced, and post-interventional TUS was established as the initial standard procedure. The driving factor for this was the previous practical and teaching experience of the new team, based on the newly introduced pneumothorax guideline in Germany [19]. Thus, from April 2017 to December 2020 both methods were used with a preference to TUS (period B). Selection of the method to be used was at the discretion of the examining physician. Interventions without a risk of PTX and those with PTX exclusion by computed tomography (CT) were excluded, as well as TUS not performed by the pulmonology team. TUS was performed by the examining pulmonologist directly after the intervention. CR was performed in the Department of Radiology and assessed by a radiologist. CR images were taken in the upright position whenever the patient’s general health allowed it with a delay of at least two hours. In order to verify the reduction in CRs done for post-interventional PTX exclusion, both periods were compared. For the comparison of both methods, CR and TUS, only period B was considered.

### Data collection

The schedules of the bronchoscopy and ultrasound units were retrospectively screened for all interventions requiring image control. Digital patient records were used to collect biometric and demographic data, data on the type of intervention, the imaging procedure used, the respective PTX therapy and its outcome. Furthermore, for both imaging modalities, instances of erroneously ruled-out PTX were noted.

### Types of interventions

**Fig. 1:**
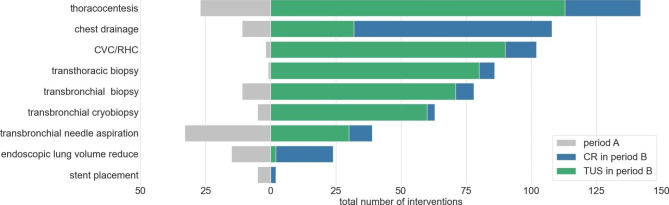
Types and frequency of interventions and their methods for pneumothorax exclusion. CVC/RHC, central venous catheter/right-heart catheterization. CR, chest radiography. TUS, thoracic lung ultrasound

In order to reflect the real-world setting, all interventions with the risk of post-interventional PTX were included in the analysis (Fig. 1). Interventional bronchoscopies were executed as needed: Both flexible and rigid inspection was possible. All interventions were performed as advised in the respective guidelines: Transbronchial biopsy (TBB) of interstitial lung disease or potential neoplastic formations using forceps, needle or cryoprobe, endoscopic lung volume reduction (EVLR) using valves, endobronchial ultrasound (EBUS) when hilar lymph node stations or intraparenchymal lesions were punctured, transthoracic biopsy of pleural lesions and pulmonary consolidations. Interventions with stent placement received imaging only when recanalization was part of the procedure. Chest tubes included both, short-term drainage with various diameters as well as indwelling pleural catheters. For chest tubes, PTX was only recorded as a complication if it required additional treatment as small post-interventional and asymptomatic PTX are intrinsic to this type of intervention. Central venous catheters (CVC) and right-heart catheterization (RHC) were combined into one group due to their similar risk of PTX and similar site of intervention. In both interventions, PTX had to be excluded only if access was via the jugular or subclavian vein.

### Ultrasound examination and PTX therapy

In the month leading up to defining TUS as the standard, TUS was taught to a team of several attending physicians and residents by an attending physician who was proficient in the method. Initially, TUS was performed under supervision during routine care, later the attending physician who had taught the method was consulted only when there were inconclusive findings.

TUS was performed by or supervised by a trained examiner with the patient in supine or semi-erect position, in accordance with the guideline recommendation [19, 20] and earlier recommendations for TUS [21, 22]: TUS was performed bilaterally either using a 13–5 MHz linear or a 5–1 MHz convex probe (Hitachi Arietta V70, FUJIFILM Healthcare, formerly Hitachi Medical Corp., Japan) without a specific thorax/lung preset. Alternatively, handheld portable ultrasound devices (i.e. Butterfly iQ, Butterfly Network Inc., USA) were used. Detection of one of the following pleural integrity features constituted PTX exclusion: pleural sliding [23], B-Lines [24] or lung pulse [25]. If all of these signs were absent, the lung point [26], as proof of PTX, was searched by laterally moving along the thorax in the intercostal space. Total PTX was assumed if unilaterally neither pleural sliding nor lung point were detectable. Use of the M-Mode to record the barcode sign was left to the discretion of the examining physician.

Clinically stable patients with PTX were monitored using TUS, in symptomatic patients a small diameter chest tube was inserted. In case of diagnostic uncertainty, a further check was performed on the same day at the discretion of the examining physician. CR was used as the primary method only when the CR would yield additional information (e.g. intrathoracic position of the chest drain tip), sufficient competence regarding the use and interpretation of TUS was not present, or in case of missing ultrasound facilities.

### Missing data and statistical analysis

Interventions were excluded from the dataset when either the imaging method or its result were unclear or when patient files were ambiguous. Interventions with missing data on body weight or height were included and data was labelled as missing. The data were analyzed using SPSS Statistics (IBM, version 28.0, New York, United States). Categorical data were presented using absolute and relative frequencies, and metric data using mean and standard deviation. The chi-square test was used to check the reduction in the number of CRs after the introduction of TUS.

## Results

### Inclusions and exclusions

By screening intervention schedules, 871 interventions requiring PTX exclusion were found. 7 of these were excluded from the data set because the primary PTX exclusion was performed by CT. Another 110 interventions were excluded because they were performed by medical departments other than pneumology. A total of 754 interventions were included, with 110 (14.6%) performed in period A and 644 (85.4%) performed in period B.

### Baseline characteristics

Baseline characteristics of the CR and the TUS group in period B are compared in Table 1.

**Table 1:** Characteristics of the CR and the TUS group in period B

	CR (n = 166)	TUS (n = 478)	p-value
age (years)	66.46 ± 12.59	65.62 ± 13.79	0.489
sex			
male	98 (59.0)	273 (57.1)	0.666*
female	68 (41.0)	205 (42.9)	
body weight (kg)	74.59 ± 18.82	77.24 ± 18.64	0.122
body height (cm)	171.48 ± 8.66	170.57 ± 9.03	0.259
BMI (kg/m²)	25.26 ± 5.8	26.54 ± 6.2	0.020

Data are expressed as mean ± standard deviation or number (%) of patients. * Based on chi²-test, other p-values obtained from Student’s t-test. BMI, body mass index. CR, chest radiography. TUS, thoracic lung ultrasound.

**Fig. 2:**
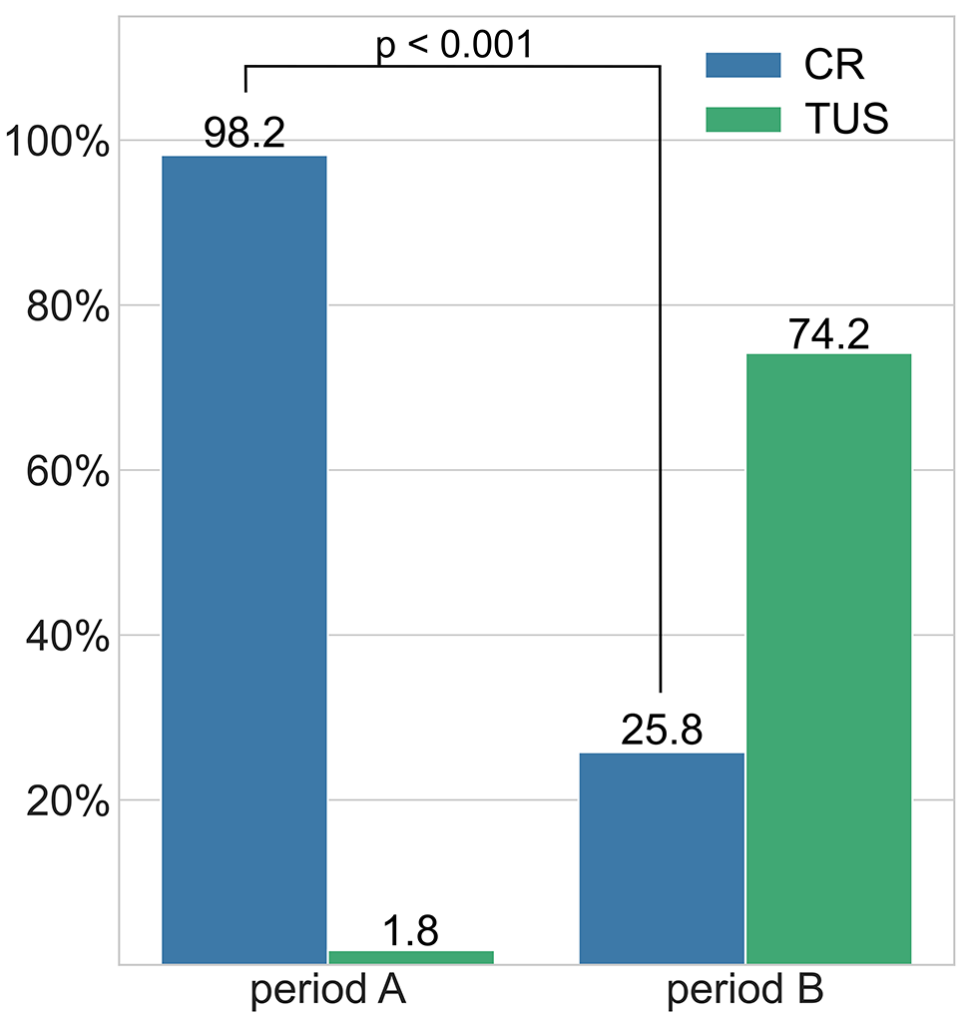
Distribution of chest radiography (CR) and thoracic lung ultrasound (TUS) in periods A and B

Details on intervention numbers, diagnosed and missed PTX, as well as investigations done to confirm initial findings are provided in Table 2. Figure 2 reflects the shift in relative frequency of CR and TUS. The relative frequency of CR decreased from 98.2% (n = 108) in period A to 25.8% (n = 166) in period B (p < 0.001).

**Table 2:** Main results in period A and B

	period A				period B		
	CR	TUS	total		CR	TUS	total
interventions	108 (98.2)	2 (1.8)	110 (100)		166 (25.8)	478 (74.2)	644 (100)
pneumothorax							
initially diagnosed	5 (4.6)	*	5 (4.5)		14 (8.4)	14 (2.9)	28 (4.3)
initially missed	*	*	*		*	1 (0.2)	1 (0.2)
confirmatory investigations							
total	*	*	*		3 (1.8)	21 (4.4)	24 (3.7)
ultrasound	*	*	*		*	1 (0.2)	1 (0.2)
chest radiography	*	*	*		2 (1.2)	19 (4.0)	21 (3.3)
computed tomography	*	*	*		1 (0.6)	1 (0.2)	2 (0.3)

Data are reported as number (%) of patients. When the number was 0, it is marked with an asterisk (*). CR, chest radiography. TUS, thoracic lung ultrasound.

Individual types and frequencies of interventions together with their methods for PTX exclusion are shown in Fig. 1. TUS was used almost exclusively for PTX exclusion in the following interventions: thoracocentesis, central venous catheter/right-heart catheterization, transthoracic and transbronchial biopsy, transbronchial cryobiopsy and transbronchial needle-aspiration. In contrast, CR was dominantly used in cases of chest drainage, endoscopic lung volume reduction and stent placement.

### Confirmatory investigations

No confirmatory investigations were documented in period A (Table 2). A total of 24 (3.7%) were conducted in period B. For TUS, 21 (4.4%) interventions prompted a confirmatory investigation. 19 were performed by CR and one each by TUS or CT. For CR, there were three confirmatory investigations out of 166 (1.8%). Of these, two were performed using CR and one using CT. Confirmatory examinations revealed one missed PTX for TUS and none for CR .

### Diagnosed PTX, missed PTX and PTX treatment

**Fig. 3:**
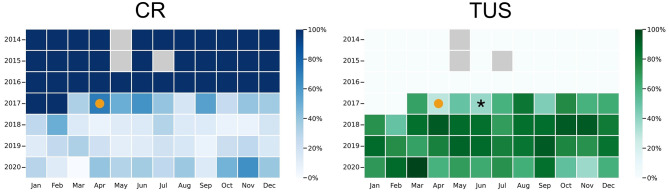
Distribution of post-interventional thoracic lung ultrasound (TUS) and chest radiography (CR) for pneumothorax detection over the duration of the study. Left panel: Monthly fraction of pneumothorax (PTX) exclusions by chest radiograph. Right panel: Fraction of PTX exclusions conducted using ultrasound. The orange dot marks the introduction of TUS as the standard. The tile with an asterisk (*) indicates a month in which one pneumothorax was missed by the initial imaging. Gray tiles indicate months without interventions requiring post-interventional PTX exclusion

A total of 34 (4.5%) PTX were diagnosed in 754 interventions (Table 2). In period A, 5 (4.6%) PTX were diagnosed, all by CR. No clinically relevant PTX was missed on initial imaging. In period B, a total of 29 (4.5%) PTX were identified. 28 were detected on initial imaging, 14 (8.4%) by CR and 14 (2.9%) by TUS. One PTX (0.2%) was initially missed by TUS after thoracocentesis, none were missed by CR. The missed PTX was small, trapped in the lung apex and had not been generated during the intervention itself. It was already described in a CT from the previous day and was not detected by TUS. It was included in the database for completeness. Treatment was conservative. The timing of the initially missed PTX in relation to the introduction of TUS is shown in Fig. 3. Of the 28 PTX considered, 15 (53.6%) were treated conservatively and 13 (46.4%) by chest tube insertion (Table 3). The treatment of the 14 PTX diagnosed by TUS was 50% conservative and 50% by chest drainage. Of the 14 PTX diagnosed by CR, 8 (57%) were treated conservatively and 6 (43%) with chest drainage. Transbronchial cryobiopsies had the highest incidence of post-interventional PTX (9.5%).

**Table 3:** Therapy of the initially diagnosed pneumothoraces in period B

intervention	total	PTX	imaging method			therapy of PTX	
			CR	TUS		conservative	chest drainage
thoracocentesis	142	6 (4.2)	5 (83.3)	1 (16.7)		4 (66.7)	2 (33.3)
transbronchial biopsy	78	5 (6.4)	1 (20.0)	4 (80.0)		3 (60.0)	2 (40.0)
transbronchial cryobiopsy	63	6 (9.5)	2 (33.3)	4 (66.7)		3 (50.0)	3 (50.0)
transthoracic biopsy	86	4 (4.7)	0 (0.0)	4 (100.0)		2 (50.0)	2 (50.0)
chest tube *	108	3 (2.8)	3 (100.0)	0 (0.0)		2 (66.7)	1 (33.3)
other interventions **	167	4 (2.4)	3 (75.0)	1 (25.0)		1 (25.0)	3 (75.0)
total	644	28 (4.3)	14 (50.0)	14 (50.0)		15 (53.6)	13 (46.4)

Data are reported as number (%) of patients. * Only PTX which received additional therapy in addition to drainage are reported. ** Other interventions included central venous catheters and right-heart catheters (1 PTX in 102 interventions), endoscopic lung volume reductions (1 PTX in 24 interventions), stent placements (0 PTX in 2 interventions) and transbronchial needle aspirations (2 PTX in 39 interventions). CR, chest radiography. PTX, pneumothorax. TUS, thoracic lung ultrasound.

## Discussion

This study demonstrates the potential and limitations of TUS when it comes to reducing the use of ionizing radiation in an interventional pulmonology department. The introduction of TUS as the method of choice reduced the number of CRs from 98.2 to 25.7% respectively.

In our study, a marked increase in overall interventions is seen in period B due to a widened professional orientation. This led to a change in the distribution of interventions performed and to an overall shift towards interventional techniques. This is in line with the overall increase in importance of interventional pulmonology in recent years [27]. The most notable changes include transthoracic pleural and lung biopsies being performed by a pulmonologist with ultrasound guidance instead of by radiologists guided by CT. In addition, right-heart catheterization was conducted within the department. The increase in transbronchial cryobiopsies can be explained by its increasing importance in the diagnosis of interstitial lung disease [28].

### CR vs. TUS in period B

No considerable differences were found between the CR and the TUS group regarding patient characteristics. Patients in the TUS group tended to have a slightly higher Body Mass Index. The preferred use of either method was not influenced by patient properties.

In most diagnostic studies, ultrasound is compared with CR in the supine position [4, 5, 8, 9]. However, the CR in our study was usually performed in the upright position, which offers better accuracy [29]. Therefore, findings from an observational study that demonstrated a reduction of CR used for PTX exclusion in day-to-day practice in an ICU setting [18] cannot be generalized to interventional pulmonology.

It is noticeable that relatively more PTX were diagnosed by CR (14 of 166, 8.4%) than by TUS (14 of 478, 2.9%) in period B. This finding is counter-intuitive since TUS is known to have a higher sensitivity than CR (79–97% vs. 40–52%) while specificity is similar [4–6]. While for most intervention types, numbers of PTX found coincide with the proportion of imaging method utilization, this is untrue for thoracocentesis and chest tubes. Here, more PTX were found using CR than using TUS while the relative usage of the methods would suggest the opposite. With a cumulative 8 PTX diagnosed by CR and one by TUS, these interventions skew the aggregated data. While speculative, we assume practical reasons for this observation: (A) Additional information (tube location, atelectasis etc.) beyond PTX exclusion was sought especially in high-risk interventions, and (B) search for the lung point is time-consuming thus nudging examiners to use CR when a PTX is suspected.

### Limitations of TUS

In period B, there were more frequent confirmatory investigations due to inconclusive findings after TUS (21 of 478, 4.4% vs. 3 of 166, 1.8% after CR ). The fact that all confirmatory investigations after TUS ruled out a PTX may be due to two reasons: (A) the subjective uncertainty when using the relatively new ultrasound technique; and (B) the added uncertainty of TUS in patients with lung conditions such as adhesions and bullae. While the first reason cannot be tested, pre-existing lung conditions are a known limiting factor of TUS: Shostak and colleagues have reported limitations of ultrasound investigations in 23% of their patients after pulmonary interventions (43 of 185) and have associated this with prior lung disease. Pleural sliding might be impaired due to emphysema, previous lung surgery, radiation exposure, and pleural adhesions. The authors have recommended to examine such patients by CR [12]. Similarly, pleural adhesions due to previous lung disease existed in 6 of 1023 (0.6%) patients examined in a study by Kreuter and colleagues. This pre-existing condition also prevented the detection of respiratory displacement of the lung and resulted in false-positive results. CRs excluded PTX in all 6 cases [11].

In transthoracic biopsies and transbronchial (cryo)biopsies as well as needle aspirations TUS was markedly favored by interventionalists in period B. These interventions combined, TUS was the method of choice in 91% (331 of 368). In these intervention types TUS offers prompt PTX rule-out and no further information (i.e. tip of chest tube) can be gained by CR.

In our study, due to the retrospective design, we cannot reproduce whether patients examined by CR received it primarily for such reasons. However, in this routine care dataset, the fraction of confirmatory investigations after TUS validates the limitations of TUS expected from prospective studies, especially by Shostak, in a much larger cohort.

In summary, pre-existing lung conditions may impede sonographic PTX detection. These limiting factors are assumed to have a higher prevalence in interventional pulmonology as compared to patients in emergency or intensive care settings in which most prospective TUS studies were conducted. However, pre-existing conditions rather result in false-positive than false-negative results: In the current relevant literature on post-interventional PTX exclusion, the false-negative rate is low and reported to be between 0 and 0.7% [8–10, 13, 14]. The patient with the missed PTX in our study constitutes one such rare false-negative event: He had a small trapped apical PTX – which cannot be easily diagnosed by TUS – along with a large malignant pleural effusion.

Additionally, the choice of imaging method is based on the intervention type, and CR was favored where additional information beyond PTX exclusion was sought: CR was used more frequently in cases of chest tube insertion and ELVR as well as stent placements (Fig. 1). Here, imaging delivers additional information beyond PTX evaluations, e.g. confirming atelectasis after ELVR, position of implanted stents, or tips of drainage catheters.

### Adoption of TUS

To this day, TUS for PTX detection – though well studied [4, 5] and recommended in guidelines [19, 30] – is still not broadly-established as a method in routine care [17]. The relative recency of the method may be a contributing factor. Seen on a monthly basis, the transition from only CR to mostly TUS was swift. Within one month, the team was able to safely perform TUS. Constant proportions of confirmatory investigations over the years support the assumption that proficiency in TUS is achieved within a short period.

The one erroneous PTX exclusion happened shortly after the introduction of TUS (Fig. 3). However, due to the methodological limitation of TUS in diagnosing clinically irrelevant small trapped PTX, there is no reason to believe that insufficient skills were a factor in this false-negative event.

Our adoption strategy is in line with training protocols from prospective studies in which physicians were trained by a mentor: For example, 2 h of training [31] or 10 TUS performed under supervision were required [32]. The assumption that TUS is quick and easy to learn is further supported by a small study by Monti et al.: The authors demonstrated that nonphysician emergency medical personnel can reliably detect PTX using sonography with a sensitivity of 96% and a specificity of 100% after a brief training session consisting of a slide show presentation, short video clips on the signs of PTX to observe in sonographic images, and an introduction to the sonographic device [33].

### Strengths and limitations

With our study, the practicability and capacity of TUS when it comes to reducing the use of CR as the standard in the routine care of an interventional pulmonology unit is evaluated for the first time. Based on a large single-center experience including all interventions typically seen in interventional pulmonology, we have demonstrated that using TUS for PTX detection can effectively reduce the amount of chest radiographs in everyday clinical practice. The main limitation of this study is the missing gold standard. Therefore, the true number of asymptomatic PTX missed by both methods is unknown, and hence we avoid using test characteristics terminology (sensitivity, specificity etc.) in conjunction with our results in this manuscript. However, it was not the aim of this study to assess test accuracy but to investigate TUS in a real-world setting.

## Conclusions

Thoracic ultrasound for post-interventional pneumothorax detection can effectively reduce the amount of chest radiographs in the routine care of an interventional pulmonology department, avoiding ionizing radiation and saving resources. The method can be implemented quickly in routine care. However, chest radiograph may still be favored when additional information is sought, or pre-existing conditions limit sonographic findings.

## Data Availability

Anonymized data is available upon reasonable request to the corresponding author.

## Abbreviations

CR: chest radiography
CT: computed tomography
CVC: central venous catheters
EBUS: endobronchial ultrasound
ELVR: endoscopic lung volume reduction
ICU: intensive care unit
PTX: pneumothorax
RHC: right-heart catheterization
TBB: transbronchial biopsy
TUS: thoracic ultrasound

## References

[1] Baumann MH, Noppen M, Pneumothorax (2004). Respirology.

[2] Celik B, Sahin E, Nadir A, Kaptanoglu M (2009). Iatrogenic pneumothorax: etiology, incidence and risk factors. Thorac Cardiovasc Surg.

[3] Cantey EP, Walter JM, Corbridge T, Barsuk JH (2016). Complications of thoracentesis: incidence, risk factors, and strategies for prevention. Curr Opin Pulm Med.

[4] Alrajab S, Youssef AM, Akkus NI, Caldito G (2013). Pleural ultrasonography versus chest radiography for the diagnosis of pneumothorax: review of the literature and meta-analysis. Crit Care.

[5] Alrajhi K, Woo MY, Vaillancourt C (2012). Test characteristics of ultrasonography for the detection of pneumothorax: a systematic review and meta-analysis. Chest.

[6] Ding W, Shen Y, Yang J, He X, Zhang M (2011). Diagnosis of pneumothorax by radiography and ultrasonography: a meta-analysis. Chest.

[7] Goodman TR, Traill ZC, Phillips AJ, Berger J, Gleeson FV (1999). Ultrasound detection of pneumothorax. Clin Radiol.

[8] Chung MJ, Goo JM, Im J-G, Cho JM, Cho SB, Kim SJ (2005). Value of high-resolution ultrasound in detecting a pneumothorax. Eur Radiol.

[9] Garofalo G, Busso M, Perotto F, De Pascale A, Fava C (2006). Ultrasound diagnosis of pneumothorax. Radiol Med.

[10] Sartori S, Tombesi P, Trevisani L, Nielsen I, Tassinari D, Abbasciano V (2007). Accuracy of transthoracic sonography in detection of pneumothorax after sonographically guided lung biopsy: prospective comparison with chest radiography. AJR Am J Roentgenol.

[11] Kreuter M, Eberhardt R, Wenz H, Schmitteckert H, Heussel CP, Herth F (2011). [Diagnostic value of transthoracic ultrasound compared to chest radiography in the detection of a post-interventional pneumothorax]. Ultraschall Med.

[12] Shostak E, Brylka D, Krepp J, Pua B, Sanders A (2013). Bedside sonography for detection of postprocedure pneumothorax. J Ultrasound Med.

[13] Galbois A, Ait-Oufella H, Baudel JL, Kofman T, Bottero J, Viennot S (2010). Pleural ultrasound compared with chest radiographic detection of pneumothorax resolution after drainage. Chest.

[14] Reissig A, Kroegel C (2005). Accuracy of transthoracic sonography in excluding post-interventional pneumothorax and hydropneumothorax. Comparison to chest radiography. Eur J Radiol.

[15] Viglietta L, Inchingolo R, Pavano C, Tomassetti S, Piciucchi S, Smargiassi A (2017). Ultrasonography for the diagnosis of Pneumothorax after Transbronchial Lung Cryobiopsy in diffuse Parenchymal Lung Diseases. Respiration.

[16] Matus I, Raja H (2019). Protocolized thoracic Ultrasonography in Transbronchial Lung Cryobiopsies: a potential role as an Exclusion Study for Pneumothorax. J Bronchol Interventional Pulmonol.

[17] Berlet T, Fehr T, Merz TM (2014). Current practice of lung ultrasonography (LUS) in the diagnosis of pneumothorax: a survey of physician sonographers in Germany. Crit Ultrasound J.

[18] Brogi E, Bignami E, Sidoti A, Shawar M, Gargani L, Vetrugno L (2017). Could the use of bedside lung ultrasound reduce the number of chest x-rays in the intensive care unit?. Cardiovasc Ultrasound.

[19] Schnell J, Beer M, Eggeling S, Gesierich W, Gottlieb J, Herth F, et al. S3-Leitlinie: Diagnostik und Therapie von Spontanpneumothorax und postinterventionellem Pneumothorax. Arbeitsgemeinschaft der Wissenschaftlichen Medizinischen Fachgesellschaften; 2018. 10.1055/a-0588-4444 30041262

[20] Buda N, Kosiak W, Wełnicki M, Skoczylas A, Olszewski R, Piotrkowski J et al. Recommendations for lung Ultrasound in Internal Medicine. Diagnostics (Basel). 2020;10(8). 10.3390/diagnostics10080597 PMC7460159 32824302

[21] Volpicelli G (2011). Sonographic diagnosis of pneumothorax. Intensive Care Med.

[22] Eisenmann S, Winantea J, Karpf-Wissel R, Funke F, Stenzel E, Taube C et al. Thoracic ultrasound for Immediate Exclusion of Pneumothorax after Interventional Bronchoscopy. J Clin Med. 2020;9(5). 10.3390/jcm9051486 PMC7291137 32429057

[23] Lichtenstein D, Menu Y (1995). A bedside ultrasound sign ruling out pneumothorax in the critically ill. Lung sliding. Chest.

[24] Lichtenstein D, Mezière G, Biderman P, Gepner A (1999). The comet-tail artifact: an ultrasound sign ruling out pneumothorax. Intensive Care Med.

[25] Lichtenstein DA, Lascols N, Prin S, Mezière G (2003). The “lung pulse”: an early ultrasound sign of complete atelectasis. Intensive Care Med.

[26] Lichtenstein D, Mezière G, Biderman P, Gepner A (2000). The “lung point”: an ultrasound sign specific to pneumothorax. Intensive Care Med.

[27] Shafiq M, Lee H, Yarmus L, Feller-Kopman D (2019). Recent advances in Interventional Pulmonology. Ann Am Thorac Soc.

[28] Lentz RJ, Argento AC, Colby TV, Rickman OB, Maldonado F (2017). Transbronchial cryobiopsy for diffuse parenchymal lung disease: a state-of-the-art review of procedural techniques, current evidence, and future challenges. J Thorac Dis.

[29] Beres RA, Goodman LR (1993). Pneumothorax: detection with upright versus decubitus radiography. Radiology.

[30] MacDuff A, Arnold A, Harvey J (2010). Management of spontaneous pneumothorax: british thoracic Society Pleural Disease Guideline 2010. Thorax.

[31] Abbasi S, Farsi D, Hafezimoghadam P, Fathi M, Zare MA (2013). Accuracy of emergency physician-performed ultrasound in detecting traumatic pneumothorax after a 2-h training course. Eur J Emerg Med.

[32] Blaivas M, Lyon M, Duggal S (2005). A prospective comparison of supine chest radiography and bedside ultrasound for the diagnosis of traumatic pneumothorax. Acad Emerg Med.

[33] Monti JD, Younggren B, Blankenship R (2009). Ultrasound detection of pneumothorax with minimally trained sonographers: a preliminary study. J Spec Oper Med.

